# Factors Affecting the Retention of Indigenous Australians in the Health Workforce: A Systematic Review

**DOI:** 10.3390/ijerph15050914

**Published:** 2018-05-04

**Authors:** Genevieve C. Lai, Emma V. Taylor, Margaret M. Haigh, Sandra C. Thompson

**Affiliations:** 1 School of Nursing & Health Studies, Georgetown University, 3700 O St. NW, Washington, DC 20057, USA; gcl29@georgetown.edu; 2 Western Australian Centre for Rural Health, The University of Western Australia, 167 Fitzgerald Street, Geraldton, WA 6530, Australia; haighm@tcd.ie (M.M.H.); sandra.thompson@uwa.edu.au (S.C.T.); 3 School of Nursing and Midwifery, The University of Dublin Trinity College, 2 Clare Street, Dublin 2, Ireland

**Keywords:** Aboriginal and Torres Strait Islander, indigenous, health personnel, health workforce, workforce development, retention, turnover intention, attrition, job satisfaction, stress

## Abstract

Indigenous Australians are under-represented in the health workforce. The shortfall in the Indigenous health workforce compounds the health disparities experienced by Indigenous Australians and places pressure on Indigenous health professionals. This systematic review aims to identify enablers and barriers to the retention of Indigenous Australians within the health workforce and to describe strategies to assist with development and retention of Indigenous health professionals after qualification. Four electronic databases were systematically searched in August 2017. Supplementary searches of relevant websites were also undertaken. Articles were screened for inclusion using pre-defined criteria and assessed for quality using the Mixed Methods Assessment Tool. Fifteen articles met the criteria for inclusion. Important factors affecting the retention of Indigenous health professionals included work environment, heavy workloads, poorly documented/understood roles and responsibilities, low salary and a perception of salary disparity, and the influence of community as both a strong personal motivator and source of stress when work/life boundaries could not be maintained. Evidence suggests that retention of Indigenous health professionals will be improved through building supportive and culturally safe workplaces; clearly documenting and communicating roles, scope of practice and responsibilities; and ensuring that employees are appropriately supported and remunerated. The absence of intervention studies highlights the need for deliberative interventions that rigorously evaluate all aspects of implementation of relevant workforce, health service policy, and practice change.

## 1. Introduction

The health disparity between Aboriginal and Torres Strait Islander peoples (hereafter Indigenous Australians) and non-Indigenous Australians has been widely discussed in the literature for decades [1,2,3]. There is a growing recognition of the multitude of factors that contribute to the poorer health status of Indigenous Australians, including differences in the social determinants of health, availability and accessibility of culturally appropriate health services, and other biomedical, behavioural, and environmental factors [4]. Barriers to the use of health services by Indigenous people include fear or lack of trust of mainstream health facilities and lack of understanding or respect shown by health care providers [5,6]. This has prompted advocacy for a greater Indigenous representation in the health workforce to improve the cultural security of care and potentially help non-Indigenous clinicians provide more culturally appropriate services [5,6,7,8].

Considerable research has shown that Indigenous people want support from Indigenous staff and clinicians as part of their health care [9,10,11]. Indigenous doctors, nurses, allied health professionals, and health workers (hereafter collectively referred to as Indigenous health professionals or the Indigenous health workforce) are cultural brokers that help Indigenous patients feel more comfortable and safe throughout the delivery of care; for this reason, having Indigenous health professionals helps minimise discharges against medical advice [9]. Indigenous health professionals have diverse roles in the provision of clinical services, health promotion, and leadership within their communities [12] and often encounter their patients or clients outside of their clinical settings. This means they are able to utilise both their health knowledge and their connections to that community to help those who need health care overcome cultural and communication barriers to accessing that care [13]. Indigenous health professionals can offer important inputs at different levels and sectors within the health care system, providing care, policy and stimulating others to engage in system reform for better care. This input is relevant across the health system, from tertiary hospitals, to rural hospitals, to primary health care settings. Indigenous health professionals may be particularly important in more remote settings given the higher proportion of Indigenous people there [14] and because language barriers, miscommunication and cultural dissonance are likely to be experienced more profoundly in populations with less familiarity with and trust in Western health care [11].

Indigenous Australians are under-represented in the health workforce, and in 2011 comprised only 1.6% of the health workforce, although they account for 3% of the population and a higher proportion of patient populations [14,15]. Large disparities between rates of Indigenous and non-Indigenous employees exist for every health profession, including nurses (in 2015, 3187 nurses and midwives identified as Indigenous, representing 1.1% of all employed nurses and midwives in Australia) and medical practitioners (0.5% of all employed medical practitioners identified as Indigenous) [16,17,18]. In 2011, Indigenous Health Workers (IHWs) represented the third largest occupation of Indigenous health professionals (behind nurses and nursing support and personal care workers); accounting for 17% of the Indigenous health workforce and 0.2% of the entire health workforce [15,19]. An IHW is defined as an Aboriginal and/or Torres Strait Islander person who holds a minimum qualification of Certificate III in Aboriginal or Torres Strait Islander Primary Health Care and provides health services or health programs directly to Indigenous Australians [20]. There is a wide degree of variation in IHW position titles (examples include Mental Health Worker, Family Health Worker, Drug and Alcohol Worker, Community Worker, and Primary Health Care Practice Manager), scope of practice, and career pathways. These discrepancies have resulted in many challenges for IHWs, including difficulties obtaining accurate workforce data. However, an urgent need for more IHWs is recognised [19,21].

Despite initiatives such as *Closing the Gap*, the shortfall in the Indigenous health workforce compounds the health disparities experienced by Indigenous Australians and places additional pressure on Indigenous health professionals [22]. Staffing shortages could be partially addressed by encouraging more Indigenous Australians into health careers, but supporting existing Indigenous health professionals to remain in the health workforce long-term is equally important. Although data regarding turnover rates and turnover intention within the Indigenous health workforce is scarce, high turnover rates for IHWs occurs [19,21,23,24]. It should be noted that workforce retention is different from turnover—one is a measure of employees who stay, whereas the other measures number of completions of employment (which may reflect contract expiry without renewal, resignation, sacking, or retrenchment) [25]. The disadvantages of poor workforce retention and high turnover are clear: heavier workloads for the remaining staff, lack of continuity of services for patients (especially detrimental for management of chronic diseases, the prevalence of which is high for Indigenous Australians) and the financial costs to employers of loss of experience, vacancies and costs associated with further recruitment [15,23,24].

The aim of this systematic review is to identify literature describing the enablers and barriers to the retention of Indigenous people within the health workforce and recommendations that have been made in the existing literature. We highlight documented or proposed strategies to develop the Indigenous health workforce and assist retention.

## 2. Methods

### 2.1. Search Strategy

**Figure 1:**
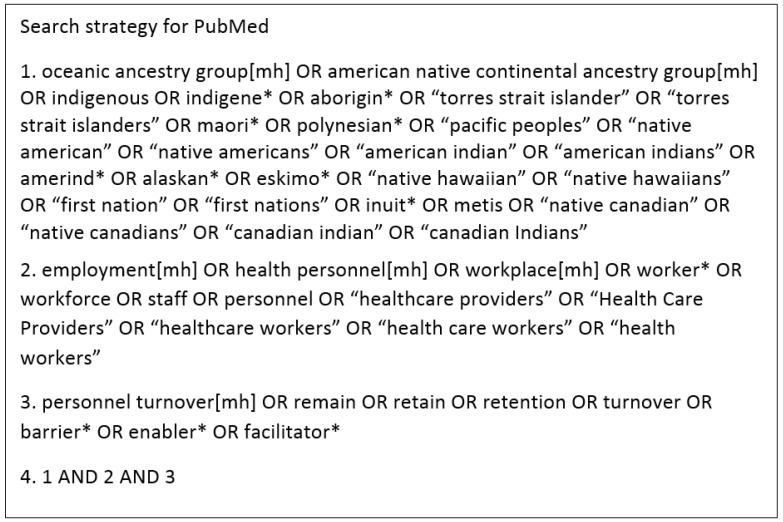
Electronic database search strategy example. Search terms varied slightly for each database.

The search was conducted in August 2017 using database-specific search strings across the following databases: PubMed, CINAHL, Informit: Indigenous Collection and Informit: Health Collection. Key search words of ‘workforce’, ‘retention’, and ‘Indigenous’ were searched using a combination of prescribed subject headings and free text keywords (see Figure 1). All search strategies are described in the Supplementary Materials. Using generic search terms, we also searched Indigenous Health*InfoNet* and an EndNote database of references supplied by Indigenous Health*InfoNet*. To identify relevant grey literature, the websites of the following organisations were searched: Health Workforce Australia (HWA), Leaders in Indigenous Medical Education (LIME), Congress of Aboriginal and Torres Strait Islander Nurses and Midwives (CATSINaM), Australian Indigenous Doctors Association (AIDA), Indigenous Allied Health Australia (IAHA), and National Aboriginal and Torres Strait Islander Health Worker Association (NATSIWA). Citation snowballing was also utilized.

### 2.2. Screening Process: Inclusion and Exclusion Criteria

We included articles that discussed supporting or inhibiting factors or recommendations relating to the retention or turnover of Indigenous Australian people in the health workforce or which discussed enablers or barriers to job satisfaction and burnout, which have clear links to retention or turnover rates in both the Indigenous and non-Indigenous health workforce [25,26,27,28]. Studies on all health professions and health workplaces were included. Two reviewers (MMH and GCL) independently screened titles and abstracts of publications identified in the initial search in relation to the following predetermined exclusion criteria: (i) language other than English, (ii) publication prior to the year 2007, (iii) Indigenous population was patients not health professionals, or (iv) not based on findings from Australia, Canada, New Zealand, or the USA. The search was limited to publications from the year 2007 onwards to capture articles from the most recent decade, which coincides with when the Council of Australian Governments (COAG) committed to ‘closing the gap’ in life expectancy between Indigenous and non-Indigenous Australians and intensified focus on Indigenous health improvement.

This review originally planned to examine Indigenous health workforce retention issues in countries sharing similar histories of colonisation and marginalisation—Australia, New Zealand, Canada, and the United States of America (USA). However, following the initial search, screening, and examination of the articles, only three articles each were identified from New Zealand and the USA, and none from Canada. The limited number of international articles identified suggested that a comparative review was unlikely to be comprehensive. Given that the underlying aim of the review was to inform workplace practices in Australia, the scope of the literature review was then limited to focus on retention enablers, barriers, and strategies related to Australia.

The search identified many articles that related to Indigenous people and support through their health training courses/degrees. While recognising that the types of pre-qualification support found to be effective in supporting students through their courses were likely to be applicable to health professionals once in the workplace, a systematic review of retention in training required a different search strategy and our primary interest was in what the literature reported on retention of trained health professionals. Hence, these articles were not within the scope of our current review and were not included.

All four authors were involved in reviewing the full texts, and a minimum of two reviewers independently reviewed and ranked each article. The reviewers developed agreed criteria for ranking articles based on relevance. Any differences in the assignment of rankings were discussed and resolved by a minimum of two reviewers. A publication was excluded following full text examination if it (i) included no findings or connection to retention or turnover related to the Indigenous health workforce (such as articles that just reported on positive or negative aspects of the workplace or profession, without any connection to retention or turnover), (ii) did not include a clearly defined Indigenous health workforce, or (iii) focused on retention in tertiary health programs or other training programs for Indigenous people not yet in the health workforce. Additionally, multiple papers from the same authors reporting on the same study population were included only if there were differences in the findings. The Preferred Reporting Items for Systematic Review and Meta-Analysis (PRISMA) statement was used with the aim of minimising methodological bias and to meet standards for accurate and consistent reporting [29].

### 2.3. Quality Appraisal and Analysis

Quality scores were calculated using the Mixed Methods Appraisal Tool (MMAT)—Version 2011 [30]. The MMAT critical appraisal tool was selected as it permits researchers to concomitantly review qualitative, quantitative and mixed methods studies (all of which were included in this review) and has been found to be efficient, reliable and has demonstrated content validity [31,32]. All articles reporting primary research were independently assessed by GCL and EVT. Any small discrepancies in MMAT scores were resolved through discussion; large variation in scoring was observed in only one article, which was referred to SCT (Sandra C. Thompson) for adjudication. Difficulties scoring two articles, where criterion 4.4 regarding response rate was not relevant, were resolved through communication with the tools authors’ [33], and the score of “Can’t tell” was assigned. Relevant data were extracted and summarised by GCL and EVT (Table 1 and Table 2).

**Figure 2:**
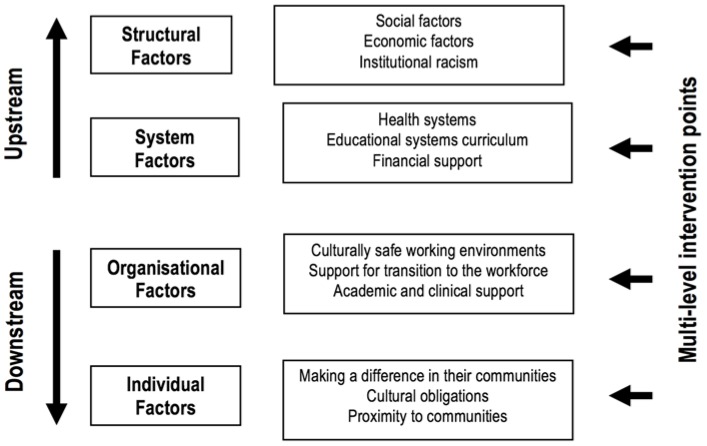
Determinants of Indigenous health workforce participation. Source: Adapted with permission from Ratima et al. [27].

Each article was reviewed, and enablers, barriers, and recommendations relating to retention were identified. These factors affecting retention were then analysed and grouped according to the following categories: (1) structural factors (such as historical, cultural, and socioeconomic), (2) health and education system factors (pertaining to overall systems, not only individual institutions), (3) organisational factors (pertaining to discrete health organisations), and (4) individual level factors (pertaining to a person’s characteristics and/or relationships). This categorization was adapted from a model of the determinants of Maori health and disability workforce participation which offered a logical hierarchy to structure the data [27] (see Figure 2). Like many frameworks, categories are not perfectly distinct, with overlap between the systems and organisational categories.

## 3. Results

**Figure 3:**
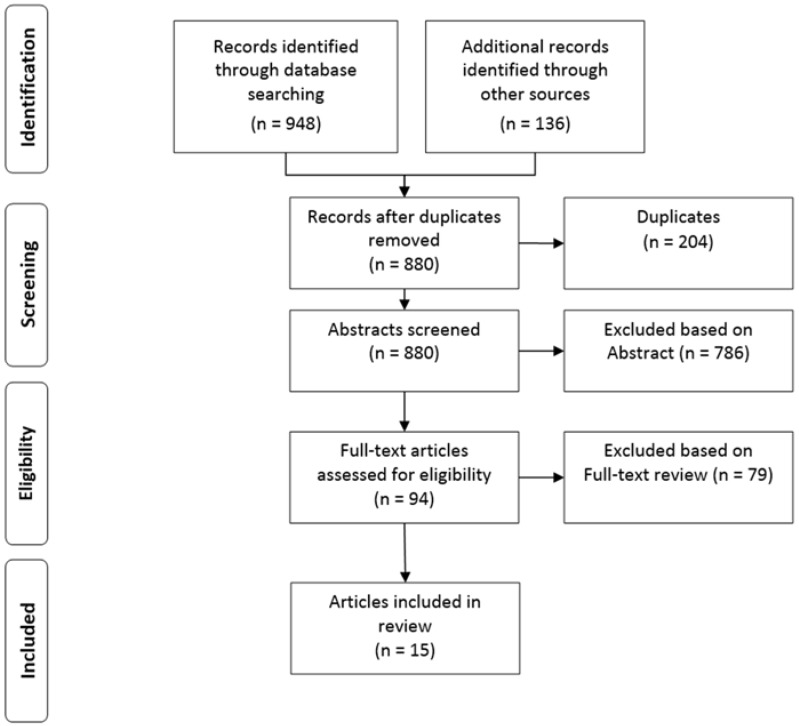
Search results and screening process based on PRISMA (Preferred Reporting Items for Systematic Review and Meta-Analysis) statement.

We identified 15 articles that met our inclusion criteria. The results for each stage of our search and screening processes are shown in the PRISMA flow diagram (Figure 3).

### 3.1. Description of Studies

The articles included in this review were studies from peer-reviewed journals (n = 10, 67%) which are outlined in Table 1 or grey literature reports from health workforce organisation websites (n = 5, 33%) which are outlined in Table 2. The majority of included articles were published since 2012 (n = 11, 73%). IHWs, Indigenous alcohol and other drug workers (IAODWs) and Indigenous mental health workers (IMHWs) were the most studied Indigenous health profession in this review, with six of the articles looking at factors affecting retention of IHWs, followed by IAODWs (n = 3) and IMHWs (n = 2). Other articles studied factors affecting the retention of Indigenous child health workers (ICHWs) (n = 1), nurses and midwives (n = 1) and Indigenous health leaders (n = 1). One article had no study population. No articles meeting the inclusion criteria reported on factors affecting the retention of Indigenous doctors or Indigenous allied health professionals.

Enablers, barriers, and recommendations for retention in the health workforce were grouped into structural, system, organisational, and individual level factors and are summarised in Table 3.

### 3.2. Enablers

No structural or system level enablers were identified in articles included in the review.

#### 3.2.1. Organisational-Level Factors

*Co-worker support and peer mentorship*. Mentoring and support from colleagues were the most frequently mentioned enablers for retention (n = 8) [19,34,38,39,40,42,46,48]. Co-worker support was found to significantly predict job satisfaction, minimize work-related strain and alleviate emotional fatigue, stress and burnout [40]. Good working relationships and peer mentorship programs provided opportunities to collaborate with Indigenous colleagues and other health workers, helped foster teamwork, partnerships and reciprocal learning, and provided support for Indigenous employees within mainstream health organisations [34,38,39,42,46,48].

*Culturally safe workplace*. Indigenous health professionals reported that culturally safe practices in their work environments, such as respect for culture, flexible working arrangements and recognition of achievements, contributed to high job satisfaction, and were important enablers of retention [41,42,47,48]. Respect for Indigenous culture by non-Indigenous health leaders allowed for empowerment and cultural safety of workers and facilitated co-worker and supervisor understanding that Indigenous health professionals may need flexible work schedules and adequate leave for community obligations [48]. Indigenous health staff appreciated being able to adjust services to meet the needs of their clients and having sufficient time to address clients’ complexities [41].

*Access to clinical and cultural supervision*. Clinical supervision and having a good supervisor were enabling factors [37,40,42,48]. Clinical supervision was reported to minimize emotional exhaustion and turnover and to result in support for staff to participate in professional development [40,42]. The presence of an Indigenous supervisor and a supportive management structure established a more welcoming and safe working environment [48].

*Professional development opportunities*. Receiving training and educational support was a facilitating factor for Indigenous health professionals [39,40,42,48]. This included on-the-job training, internal in-service courses, external courses, and opportunities to develop skills outside of their formal qualifications [48]. An article that reported on the experiences of IHWs and registered nurses (RNs) who undertook a postgraduate diabetes course found that IHWs were able to deliver diabetes education directly to their clients instead of relying on expert diabetes educators, and that the opportunity to upskill encouraged retention [39]. Financial assistance for those who participated in external courses and paid leave to attend workshops and conferences were also important facilitators [39,42].

*Job security and adequate remuneration*. Adequate salary and job security were important facilitators to retention, but only one article reported respondents being currently satisfied with their salary or benefits [40], and only two articles reported a majority of Indigenous respondents being in permanent roles [37,40].

#### 3.2.2. Individual Level Factors

*Making a difference for Indigenous health*. Being able to make a difference for their community and improve Indigenous health outcomes were identified as important contributors to job satisfaction and retention [39,41,42,47,48]. Indigenous health professionals reported feeling empowered and confident when they had opportunities to say what was needed in their community, effectively provide care for their clients and advocate for their clients’ needs [39,41,48].

### 3.3. Barriers

#### 3.3.1. Structural-Level Factors

*Racism*. Racism and stigma were major barriers faced by Indigenous health professionals [35,40,41,47,48]. Racism was reported as a significant factor in poor mental health, and racism from co-workers and a lack of respect for culturally appropriate practices led to a culturally unsafe work environment and burnout [40,41].

#### 3.3.2. System-Level Factors

*Limited organisational funding and inadequate remuneration*. Low, short-term, and non-recurrent funding of organisations and resultant job insecurity and low salary were frequently mentioned barriers (n = 8) to the retention of Indigenous health professionals [9,36,37,40,41,42,47,48]. Low pay rates reflected short-term and unstable funding to organisations especially in the non-governmental sector; this led to unpaid overtime with adverse impacts on work/life balance and subsequent burnout [37,40]. Significant pay differences were also reported between Indigenous health professionals and other health professionals with similar roles [36,40,48]. In one study, Indigenous status was found to be a significant predictor of salary, and IHWs were significantly more likely to have lower wages than other occupational groups [40]. The feeling of being inadequately compensated for a stressful job and heavy workload resulted in health professionals moving to other locations or careers with better pay [36,37,41].

*Limited career pathways*. Limited career pathways for IHWs compounded retention challenges, causing some health workers to look for work outside the health sector, where they perceived greater opportunities for career progression [36,48]. The limited career pathways were compounded by inflexibility within the tertiary education system, which prevented IHWs from easily transitioning into a formally accredited health profession. IHWs reported frustration over lack of recognition of prior learning or skills as this inhibited qualifying for certain courses or gaining unit exemptions when they chose to pursue degree courses such as nursing [36,48].

#### 3.3.3. Organisational-Level Factors

*Heavy workloads and demands*. Heavy workloads and stress, often caused by lack of role clarity or staffing shortages, were the most mentioned (n = 10) barriers to retention [9,35,36,37,38,40,41,42,47,48]. Due to shortages in resources and staff, IHWs in particular reported feeling overworked and under constant pressure as they juggled multiple roles, in some cases effectively taking on responsibilities of being the social worker, carer, community advocate, and counsellor [9,35,37,38,41,42]. There were often expectations of health workers to handle duties outside of their qualifications and experience, such as addressing issues with their clients’ housing, finances, and mental health [9,35,37]. Often, they were expected to be responsible for anything ‘Indigenous’ and would be handed responsibility for all the Indigenous clients based on an assumption that all Indigenous clients wanted Indigenous caseworkers [36,41]. Role ambiguity also led to assumptions that the scope of IHWs was primarily only a cultural role focused on “wellbeing work” and not any clinical work; thus, IHWs were often excluded from decision-making processes by other staff, resulting in burnout, job dissatisfaction, and attrition [38].

*Lack of support from management and lack of mentoring*. Lack of formal mentoring programs, clinical and cultural supervision, and poor support from management were frequently mentioned barriers to retention (n = 8). This was true both for younger Indigenous workers, who wanted guidance from older Indigenous role models, and for Indigenous health staff generally, who wanted support to strengthen their coping skills in response to stress [34,35,37,38,41,46,47,48]. Furthermore, excessive workloads, stress, and isolation experienced by Indigenous health professionals, especially in remote geographic areas, further intensified the need for support from management [35]. The lack of adequate mentoring and support systems led to low levels of job satisfaction, negative perceptions of the work environment, emotional exhaustion, and high turnover [35,38,41,46]. The absence of cultural supervision meant Indigenous health staff felt that they were not working in a culturally safe environment, contributing to retention issues [46,47].

*Lack of professional development opportunities*. Indigenous health professionals reported that the lack of opportunities and funding for further training or professional development limited pathways for career advancement [9,36,37,38,41]. Health professionals described feeling that they were underutilised, and this has been reported as contributing to increased turnover in some instances [9,36,38,41].

#### 3.3.4. Individual-Level Factors

*Proximity to community*. Eight articles described how difficulties setting culturally appropriate boundaries caused Indigenous health professionals to feel overworked and experience significant family/work life imbalance [35,36,37,40,41,42,47,48]. Strong cultural obligations to meet community expectations and being continuously available were key factors in stress and emotional exhaustion, contributing to increased turnover [40,41]. This stress was exacerbated when relatives or friends became clients, resulting in conflict between professional and community responsibilities [36,41,42].

### 3.4. Recommendations

The articles included contained no recommendations to be implemented at a structural level.

#### 3.4.1. System-Level Recommendations

*Recognition of the Indigenous health professional role*. Many articles included recommendations to improve the overall legitimacy, credibility, and support of the Indigenous health workforce, particularly IHWs (n = 8) [9,19,37,38,40,41,45,47]. At a national level, there were strong recommendations to ensure that the role of IHWs is understood and valued and to reduce confusion about the role among managers and other health professionals [19,45,47]. In order to improve IHW workforce planning and better understand retention and turnover rates, establishment of a national dataset on the IHW workforce was recommended [19,45]. At the organisation level, as part of the *Growing our future: the Aboriginal and Torres Strait Islander health worker project final report*, the HWA created the *HWA Health Service Toolkit* (the Toolkit), which aimed to “promote simple, low-cost actions that can potentially be undertaken by health services … at the local level” to support and empower IHWs [19] (p. 102). The Toolkit outlined six strategies for change with accompanying actions and contains an implementation template for health services. Action 2.1 in the Toolkit recommended that health services “collaboratively assess the role and scope of practice of [IHWs], raise awareness with other staff and clearly delineate roles and responsibilities” [19] (p. 105). Such an approach aimed to help manage employee, employer, and community expectations and to reduce heavy workloads caused by ambiguous or poorly defined job descriptions and responsibilities [9,37,38,41]. Recommendations were also made to increase the overall size of the Indigenous health workforce with the aim of reducing the heavy workloads and stress experienced by the existing workforce [19,41,45]. Creation of career pathways beyond the currently limited options was also seen as a priority for improving retention [19,40,45].

*Increased remuneration and salary parity*. Salary increases, particularly to achieve parity among Indigenous and non-Indigenous workers with similar job descriptions, and improved conditions were described as pivotal retention strategies [9,19,37,39,40,41,48]. The *Growing our future: The Aboriginal and Torres Strait Islander health worker project final report* recommended that at the organisational level health employers should review IHW salaries to ensure Indigenous staff are being appropriately remunerated. They reported that some employers used innovative business models or negotiate with funding bodies to ensure appropriate budgets to provide appropriate remuneration [19]. It was also recommended that the Indigenous health workforce consider working together across disciplines to address salary and award structures to overcome their comparatively low numbers and distribution over a vast geographic area [19,37].

*Work with educational systems to improve curriculum structure and facilitate career progression*. Retention of the Indigenous health workforce could potentially be improved by restructuring courses in tertiary health programs to increase the preparedness of students entering the health workforce and by support for career opportunities post-study [19,36,39,45]. Recommendations included expanding the variety of clinical placements undertaken during IHW training to include both Aboriginal Community-Controlled Health Services and acute care services, developing articulation pathways for IHWs who wish to undertake tertiary health qualifications, increasing the flexibility and availability of training and funding to support training, restructuring all health curriculums to incorporate principles of Indigenous learning, and involving Indigenous elders to facilitate the learning of Indigenous students [19,39,45]. An article that examined factors affecting job satisfaction of IMHWs recommended changing the Bachelor of Health Sciences degree for the IMHW training program to a recognized professional qualification such as nursing or social work, with the aim of increasing remuneration and career opportunities for IMHWs [36].

#### 3.4.2. Organisational-Level Recommendations

*Implement mentoring*, *clinical supervision and support systems*. Over half the articles (n = 9) recommended the implementation of clinical and cultural mentoring, provision of clinical supervision and improved support structures for Indigenous health professionals, to increase retention rates [9,19,34,35,37,39,41,42,45,46]. The provision of clinical and cultural mentoring for workers can increase clinical and cultural skills, develop cultural safety among non-Indigenous health professionals (based on mentoring which is two-way [34]) and provide opportunities to debrief, thus supporting emotional wellbeing and helping to reduce stress and burnout [19,34,37,41]. It was noted that senior Indigenous health professionals and non-Indigenous health professionals need to be trained in how best to provide effective and culturally safe mentoring [19,34,46]. Provision of culturally appropriate clinical supervision was highlighted as important and as having the potential to increase job satisfaction, retention and improve client outcomes [41,45]. Cultural supervision was also recommended, and it was suggested that workplaces without the capacity to offer cultural supervision hire an external cultural mentor to support the Indigenous health staff [37]. Supervisors supporting Indigenous health professionals to connect with other Indigenous health staff, can facilitate building support networks that contribute to retention [9].

*Embed cultural respect in the workplace*. Recommendations to embed cultural respect for Indigenous Australians were repeatedly mentioned [19,34,36,37,41,45,47]. Reports have recommended that health staff at all levels receive ongoing cultural safety training, with completion of training embedded in performance management requirements [19,45,47]. Such training has the potential to increase understanding of the IHW role and improve collaboration and respect between IHWs and other health professionals, empowering Indigenous health professionals and reducing burnout [19,36,37,41]. Moreover, improving the cultural competency of health leaders and supervisors may help create more culturally sensitive workplace environments, encourage consultation with Indigenous staff regarding health policies in their communities and promote ‘Indigenous ways of working’ with clients [40,41].

*Professional development opportunities*. Support for ongoing professional development to strengthen the clinical and non-clinical skills and capabilities of the Indigenous health workforce across all health disciplines was recommended as important to improve retention and facilitate progression in career pathways within the organisation [19,39,40,41,42,45,47]. Practical recommendations to support professional development included ensuring that all Indigenous health professionals have a Continuing Professional Development (CPD) plan to achieve their professional goals, with leave approved for them to attend training and provision of backfill for their position if required, having work time allocated for study purposes or subsidising training costs where appropriate, providing opportunities to implement new learnings in the workplace after the completion of training, and recognising increased qualifications gained by changes to an employee’s role description and appropriate remuneration for postgraduate qualifications [19,39,47]. One article suggested that specific training in assertiveness and ‘boundary-setting’ was important to help Indigenous health professionals set culturally appropriate boundaries and manage community expectations [41].

*Flexible working arrangements*. Flexible employment arrangements that allowed workers to meet both their professional and community obligations were recommended to improve work/life balance and increase retention [19,40,41]. This included adequate leave provisions for cultural commitments such as sorry business [19,41].

## 4. Discussion

This paper set out to explore issues identified in the literature associated with retaining Indigenous health staff in the workforce. The original search strategy sought to identify literature examining Indigenous health workforce retention issues from New Zealand, Canada, and the USA, as well as Australia. Due to the small number of international articles remaining following the screening of the full-texts, the initial proposal for a comparative piece between the four countries was not progressed as it was considered unlikely to be comprehensive. We subsequently learned that in the USA, measures to address the under-representation of Indigenous peoples in the professional health workforce are generally subsumed within wider initiatives that target African Americans and other ethnic minorities [27] such that relevant articles may not have been retrieved by the Indigenous-specific search terms used. One-third of the relevant literature for Australia was grey literature identified by searching government and health professional organisations’ websites known to the Australian authors, indicating that relevant Indigenous health information does not always get published in the peer-reviewed literature. Unfortunately, the authors are less familiar with the relevant websites for New Zealand, Canada, and the USA, constraining our approach to reliable identification of relevant grey literature. However, it is likely that Australia could learn from collaborative work with relevant agencies in these countries, and we recommend such work be undertaken in future.

We identified only 15 articles that discussed supporting or inhibiting factors or that made recommendations relating to Indigenous health professional retention. Despite the pressing need for Indigenous health professionals, there was a dearth of published strategies to improve retention, no documented evaluation of these strategies and no intervention trials. Far more has been written about barriers to retention than enablers, possibly reflecting a national policy emphasis on removing barriers instead of empowering Indigenous Australians, and there is limited evidence about which retention strategies are most effective [20]. The lack of national, up-to-date data about the Indigenous health workforce adds to challenges in understanding the size of the workforce and the scope of retention issues and to measuring changes in the workforce over time.

The work environment (including the attitude of health leaders and co-workers and access to supervision, mentoring, and training) was found to be a fundamental predictor of retention for the Indigenous health workforce. This accords with the findings of a previous review on IHWs [49], as well as findings about the retention of Indigenous Australians in other sectors [50,51] and on retention of non-Indigenous health workers [52,53]. A supportive workplace (including supportive co-workers, respect for Indigenous culture, and access to clinical and cultural supervision and mentoring) was found to significantly predict job satisfaction, minimize work-related strain and alleviate emotional fatigue, stress, and burnout, all of which are linked to improved retention [19,34,37,38,39,40,41,42,46,47,48]. Conversely, a workplace where racism was tolerated, or where there was limited support from management, lack of mentoring or supervision and few opportunities for training led to low levels of job satisfaction, negative perceptions of the work environment, emotional exhaustion and high turnover [9,34,35,36,37,38,40,41,46,47,48].

To achieve supportive and culturally safe workplaces, cultural respect for Indigenous Australians must be embedded across the health system [54,55]. It has been recommended that health services identify a cultural safety framework or toolkit that is relevant to their organisation and make a commitment to its implementation [19]. Reports strongly recommend that health staff at all levels receive mandatory ongoing cultural safety training, with completion embedded into performance management and/or professional development requirements [19,45,47]. Improving the cultural competency of non-Indigenous staff at all levels is expected to help to create more culturally sensitive workplace environments, and improve respect for and understanding of Indigenous health professionals, helping to empower Indigenous health professionals in the workplace and reduce burnout [36,37,40,41,48,49]. At the organisational level, workplaces can ensure that Indigenous health professionals have access to culturally-appropriate clinical and cultural supervision and can implement mentoring programs, all of which can contribute to improved job satisfaction, retention and improved client outcomes [9,19,34,35,37,39,41,42,45,46]. Workplaces can also ensure Indigenous health professionals are supported in their ongoing professional development, have clearly defined career pathways and have access to flexible working arrangements, all of which are linked with improved retention [19,39,40,41,42,45,47,49].

A study on nurses in the Northern Territory found that a supportive workplace may reduce turnover by helping mitigate some of the emotional exhaustion caused by heavy workloads [56], which was a key predictor of turnover intention for Indigenous health professionals [40]. Causes of heavy workloads in the Indigenous health workforce included lack of support from management, shortages in resources and staff, and role ambiguity, particularly for IHWs [9,35,37,38,41,42]. Lack of clear role descriptions led to IHWs feeling overworked and under constant pressure as they juggled multiple responsibilities or handled duties outside of their qualifications or experience [9,35,37]. Clearly documented roles, scope of practice and responsibilities help empower IHWs in their roles, manage their workloads, clarify employee, employer and community expectations and encourage productive working relationships, all of which are reported as contributing positively to retention [9,19,37,38,41,57]. Tools such as *The National Framework for Determining Scope of Practice for the Aboriginal and/or Torres Strait Islander Health Worker and Health Practitioner workforce* exist to help employers work with their IHWs to establish and define their scope of practice [57].

Raising awareness of, and improving respect for, the Indigenous health workforce, particularly the IHW workforce, may also cause employers to re-examine IHW remuneration and employment conditions. There is a widespread perception amongst the Indigenous health workforce, particularly IHWs, that they are being inadequately compensated for a stressful job and heavy workload, often with high levels of unpaid overtime, and that they are paid less than other health professionals with similar roles [9,19,36,37,40,41,42,47]. This was one of the most frequently mentioned barriers to the retention of Indigenous health professionals, as employees moved to other positions, or left the health sector entirely seeking higher pay and less stress [36,37,41]. This reflects findings about the retention of non-Indigenous health workers, where low salary adversely affects retention [58], and also aligns with findings about Indigenous Australians working in other sectors [50,51]. At a system level, it has been recognised for some time that there needs to be a fundamental shift in the way Indigenous health services are funded, with more and longer-term funding of health programs and positions required to improve the salary levels and job security of staff providing services [59,60]. However, few articles made practical recommendations to address this barrier beyond noting that strategies to address salary parity were urgently needed [40,41]. The concerns around salary issues were clear from the literature and were largely based on studies involving Indigenous health professionals with certificate (not university) qualification(s). The under-valuing of Indigenous staff, regardless of qualifications and experience, may be more entrenched when they work in health services focused on the general population and/or provision of specialist technical expertise, as Indigenous patients generally represent only a small proportion of overall patients [9]. Budgetary pressures on health services and reliance on historical budgets may also contribute to low salaries. More studies that showcase successful ways of working with Indigenous staff and dedicated intervention studies that document the benefits and cost-benefits from strategic development in Indigenous workforce recruitment and retention are needed.

Another cause of heavy workloads, staffing shortages, can be partially addressed by improving retention but also by encouraging more Indigenous Australians into health careers [8,12]. Interestingly, the search strategy identified many articles that referred to retention of Indigenous students as they progressed through higher education to become health professionals and identified this as an important part of retention in the workforce. There was considerable overlap in some of the enablers and barriers to retention in these two related but different settings, but our search terms were not broad enough to have captured all the relevant literature related to retention in tertiary settings, reflecting our focus on retention of the trained health workforce. Given the importance of retention in the tertiary education setting to ensuring Indigenous students graduate with health degrees, the importance of growing Indigenous health graduates across multiple disciplines and the importance of preparing graduates for their ongoing careers and the potential challenges they may face in the future, it is recommended that a systematic review of published literature in this area be undertaken. To grow and strengthen the Indigenous health workforce, workforce development policies require the inclusion of a broad range of strategies to improve Indigenous educational attainment at school, encourage enrolment into vocational and health-related training programs, support completion of the training programs, recruit Indigenous people to the health workforce, and support them to remain in the health workforce long-term [12,27]. The current review focused only on the last point, but considerable experience exists in the university sector that could usefully inform other areas of the Indigenous health workforce pipeline.

**Figure 4:**
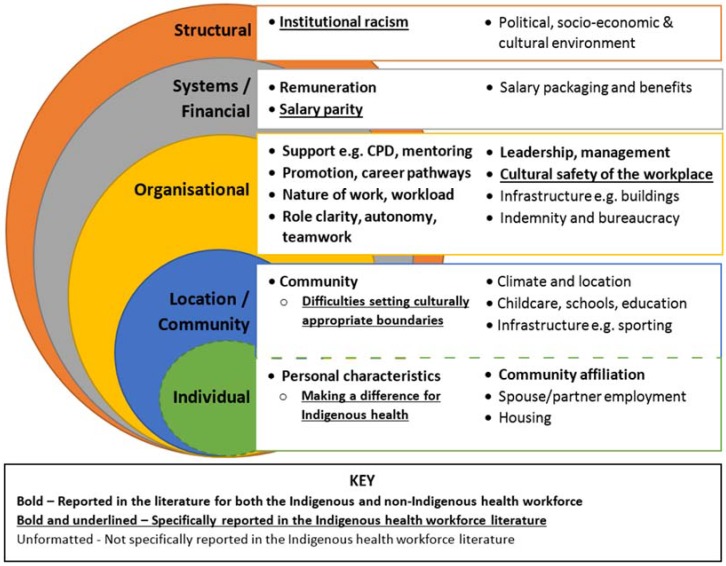
Factors affecting retention for Indigenous and non-Indigenous health professionals. Source: Based on Humphreys et al. [25] (with permission) and adapted to highlight similarities and differences for the Indigenous and non-Indigenous health workforce based upon the literature.

While some of the identified factors affecting retention concurred with the broader literature on health workforce retention, such as the importance of work environment, workload, salary and professional development opportunities [25,52,58,61,62,63], studies specific to Indigenous health professionals focused on those issues seen as more specific to them. Issues of racism, wage disparities and the influence of community both as a strong personal motivator and as a source of stress when work/life boundaries could not be maintained emerged [40,41,51]. Similarities and differences between Indigenous and non-Indigenous health professionals in factors reported to affect retention are highlighted in Figure 4, which captures the considerable overlap between the factors affecting retention [25]. However, many of the location/community and individual factors that influence retention (such as spouse/partner employment, climate and location, and infrastructure) were not specifically mentioned in the literature on Indigenous health professionals. This may reflect that Indigenous health workers, the major group reported on in the studies meeting our criteria, are more likely to come from and live locally long-term. While many of these factors likely do affect retention, they may not be elucidated when Indigenous health professionals are asked to reflect on their own experience because they are not specific to Indigenous identity or are so common for Indigenous people and not specific to working in health. Whether the pressures faced by Indigenous health professionals are different and how these can be effectively addressed requires further study.

The notable dearth of studies on the retention of university-trained Indigenous clinicians (such as Indigenous doctors, nurses or allied health professionals) was unexpected; in two-thirds of the articles respondents were IHWs, AODWs, or IMHWs. It is possible that turnover for Indigenous clinicians, who have mastered additional hurdles in being accepted into and progressing through degree qualifications at university, is lower than for IHWs who complete certificate qualifications. Completing a university health professional qualification requires resilience and the ability to function a bicultural environment. The better pay, job security, and conditions received by Indigenous clinicians may help to offset some of the barriers to retention described in this paper, although anecdotally racism and organisational cultural safety remain huge issues, and it is possible that formal studies would identify other issues. The issues raised in this paper are likely to be relevant to improving the retention of Indigenous degree-trained clinicians, but more quantitative and qualitative research with the Indigenous clinical workforce is needed to determine the extent to which retention of these professionals is a factor and to explore whether or not factors influencing retention are shared across the range of health disciplines and health settings. The cost-benefits of different intervention strategies to improve retention would also be worthy of study.

The commitment of Australian governments to Closing the Gap has resulted in increased efforts to improve Indigenous health over the last decade [3]. Employing experienced Indigenous health professionals is an important component of providing culturally safe care to Indigenous people, which in turn is critical to improving health service access. As Indigenous people represent only a small proportion (3.0% [14]) of the Australian population, their importance in health settings includes training and support for non-Indigenous professionals in appropriate ways of working, beyond just service delivery. There is no shortage of government documents and strategies that acknowledge this, but progress has been slow. Actions have not been focused or consistent, so more concerted efforts are needed. As this review has summarised the recommendations made by the literature, we provide four high priority recommendations here. First, attention to enumerating and measuring the Indigenous health workforce in ways that are more sophisticated than total numbers is required; these measures could in turn be used as performance indicators which would signal the importance attached to this parameter and in turn make turnover, vacancies and casualisation more transparent. Second, longitudinal studies of health professionals from the time they complete training are needed to enhance our knowledge and understanding of what happens to trained Indigenous health professionals and why; these longitudinal studies should utilise mixed methods and could in turn inform specific interventions to support Indigenous health professionals in their workplaces. Third, understanding and addressing all types of exposure of Indigenous health professionals to racism in the workplace and creating culturally safe environments for them is essential. Finally, implementing peer support and mentoring arrangements for Indigenous health staff requires attention, including how to do this in ways that are both effective and efficient.

### Limitations

Systematic reviews are inevitably limited by the quality and quantity of research available for inclusion. Relevant articles were mostly descriptions of the status quo, only 10 of which were published in a peer-reviewed journal, and while they made recommendations, none had tested an intervention aimed at improving retention. The scarcity of literature related to retention of university-trained health professionals after they have graduated given they may face different issues, limits the conclusions that can be drawn in relation to them, and also comparisons across geographic and workforce settings. The lack of rigorous evaluations measuring the effectiveness of retention strategies for any health workers (not just Indigenous) in rural and remote areas of Australia has previously been identified [25,63]. One third of the identified relevant literature was grey-literature, with article quality being an issue (one article was not suitable for quality appraisal and another scored poorly on MMAT). These limitations in the literature, with reports that were observational, descriptive, and cross-sectional in nature and the lack of tailored interventions designed to bring about change, underscore the need for development of the Indigenous health workforce with robust evaluation and research in this area.

## 5. Conclusions

To build a strong Indigenous health workforce, it is important not only to recruit more Indigenous Australians into health careers, but also to support existing Indigenous health professionals to remain in the health workforce long-term. Despite the pressing need for Indigenous health professionals, there is little evidence of workplaces implementing formal strategies (such as the HWA Health Service Toolkit) [19] to improve the retention of Indigenous health professionals and no documented evaluation of these strategies; therefore, the evidence about which retention strategies are most effective is very limited. However, this review has highlighted a number of important factors in improving the retention of Indigenous health professionals, such as the importance of a supportive and culturally safe workplace (including the attitude of health leaders and co-workers and access to supervision, mentoring, and training); the need to clearly document roles, scope of practice, and responsibilities; and the need for a fundamental shift in the way Indigenous health services are funded, with more and longer-term funding of health programs and positions required to improve the salary levels and job security of the staff providing those services. While this has inherent logic, there is a need for empirical testing of the principles through dedicated initiatives that are carefully evaluated.

This review has identified a need for national, up-to-date data about the Indigenous health workforce to better understand retention and turnover rates. Research into the factors affecting the pathways for Indigenous people into health careers and the factors that influence retention of Indigenous clinicians in specific health fields is needed. There is also an urgent need for retention strategies to be formally and collaboratively designed, documented, implemented, evaluated, and reported in order to determine which retention strategies are most effective. It is likely that Australia could learn from the experience in other developed countries with Indigenous minority populations, and a collaborative approach could be a useful way to progress this.

## Figures and Tables

**Table 1:** Peer-reviewed studies.

First Author (Year) Location	Methods	Study Population and Response Rate	Focus	Relevant Findings	MMAT Score
Browne et al. (2013) [34] Victoria Rurality not Specified	Qualitative Interviews	30 mentoring program participants (17 IHWs (Indigenous Health Workers), 13 AHPs (allied health professionals)). Response rate: 86% of mentoring program participants.	Evaluate peer mentoring between IHWs and non-Indigenous allied health professionals.	Peer mentoring between Indigenous and non-Indigenous health workforce found to facilitate two-way learning, meet learning needs, and promote practice improvement. Helps address the challenging nature of the work of IHWs, which currently results in high turnover.	75%
Conway et al. (2017) [35] Australia Rural and Urban	Qualitative Interviews	5 IHWs (from five different states) Case study methodology. Mass recruitment via e-mail to 201 IHWs, followed by purposive sampling selected by key informants, due to low response rate.	Barriers and facilitators for IHWs in the workplace in providing self-management support.	Causes of stress for IHWs included: time pressures, work/life imbalance and cultural expectations, lack of mentorship, high staff turnover and culturally insensitive non-Indigenous colleagues. Stress contributed to burn out and attrition, and was more prevalent in rural and remote environments. IHWs have limited support and are excluded from decision-making by other staff, also leading to burnout and attrition. Adequate staffing and support structures are required to reverse these barriers.	75%
Cosgrave et al. (2017) [36] New South Wales Rural and Remote	Qualitative Interviews	5 IMHWs (Indigenous Mental Health Workers). Criterion sampling: participants had to meet qualifications conditions to work as CMH (community mental health) professionals and had to be currently/recently working in a rural/remote local health district in NSW. Participants recruited through group presentations. Response rate: unspecified.	Factors affecting job satisfaction and retention of IMHWs (Indigenous mental health workers).	Three main factors affecting job satisfaction were: difficulties being accepted into the team and workplace caused by lack of understanding of role, challenges with setting culturally-appropriate personal and professional boundaries and perceived salary inequalities and low salary. Inadequate remuneration was linked to turnover intention. Lack of career opportunities a motivator for leaving the health sector.	50%
Ella et al. (2015) [37] New South Wales Metro 22%, Regional 59% Rural 14%, Remote 6%	Quantitative Survey	51 Indigenous AOD (alcohol and other drug) workers. Response rate: 85% of study population. No workers declined to participate. No contact data was available for the nine workers not identified for interview.	Description of Indigenous AOD workers employed in NSW and strategies to improve retention.	Improvement of retention among Indigenous AOD workers requires implementation of professional development opportunities, improved pay and job security, greater role clarity, access to formal supervision and clinical and cultural mentoring.	100%
Harris & Robinson (2007) [38] Northern Territory Remote	Mixed methods Audit, participant observation and interviews	Audit: 30 client records across five health centres. Interviews: 52 personnel and stakeholders involved in the IMHW program (all employed IMHWs, mental health clients, non-Indigenous health professionals, key stakeholders). Response rate: unspecified. Purposive sampling.	Evaluate the “Aboriginal Mental Health Worker Program” in the NT.	Role ambiguity and unclear cultural legitimacy of IMHW practice causes stress and can lead to burnout. Geographical isolation, limited mentoring, lack of support and difficulty accessing training were linked to attrition. Greater role clarity, culturally informed mental health practices and local managerial support are essential for IMHWs to be effective members of the primary health team. Support at the supra-local level with departmental strategies, resources and partnerships are required for long term improvements and systematic change.	75% ^1^
King et al. (2012) [39] New South Wales Rural and Remote	Qualitative Interviews	17 participants, educators and managers regarding the diabetes course. (5 AHW (Aboriginal Health Worker) DEs (Diabetes Educators), 1 AHW student DE, 8 RN (registered nurse) DNEs (Diabetes Nurse Educators), 1 RN student DNE, 2 Nurse Mangers. Response rate: 86% of IHWs who attended the course, unclear for other participants. Purposive sampling.	Experiences of IHWs and RNs during and after completion of specialist diabetes training, and managerial strategies to support workers during and after training.	Completing specialist training was empowering, encouraged retention, improved service delivery for clients and was a good investment for the health service. Managers can support completion of training by allocating work time for study, providing formal support, and by making changes to role, duties and/or remuneration to reflect additional qualifications.	50%
Roche et al. (2013) [40] Australia Metropolitan Rural and Remote	Quantitative Survey	294 AOD workers (184 Indigenous, 108 non-Indigenous, 2 unknown). Response rate: unknown. Convenience sampling.	Factors that contribute to the stress levels, well-being and turnover intention of Indigenous AOD workers.	Emotional exhaustion is a key predictor of turnover intention and is caused by work/family life imbalance and lack of co-worker support. Ensuring adequate and equitable salaries, providing career development opportunities and reducing stress levels may reduce turnover intention.	75% ^2^
Roche et al. (2013) [41] Australia Metropolitan Rural and Remote	Qualitative Focus Groups	121 AOD Workers (70 Indigenous, 20 non-Indigenous, 31 unspecified). Response rate: unspecified. Purposive, maximum-variation sampling.	Indigenous AOD workers experiences and perspectives on well-being, stress and burnout and strategies to improve well-being.	Heavy workloads, lack of career opportunities, poor job security and low salaries contribute to turnover intention among IAOD workers. Other sources of workplace stress included: proximity to and expectations of communities and experiences of racism and discrimination. Workforce development strategies included: adequate remuneration, supervision and mentorship, cultural sensitivity training for non-Indigenous workers, and training in boundary setting.	75% ^3^
Taylor et al. (2009) [9] Western Australia Metropolitan	Qualitative Interviews	2 IHWs, 12 non-Indigenous health professionals, 12 Indigenous patients. Response rate: Unspecified. Purposive sampling.	Impact of and challenges faced by an IHW working in cardiac rehabilitation in a tertiary hospital.	Job dissatisfaction was caused by limitations in the IHW training for hospital settings, role ambiguity and poor role definition, poor remuneration and limited career pathways. Recommendations to improve the IHW role and retention in a hospital setting included: providing additional training in the hospital setting, supporting collaborations with other Indigenous staff, documenting clear role responsibilities, and ensuring appropriate remuneration.	50%
Watson et al. (2013) [42] Queensland	Qualitative Focus Groups	47 CHWs (child health workers) (33 Indigenous, 11 non-Indigenous, 3 mixed cultural background). Response rate: 96% of program participants.	Areas of support that are important to Indigenous and non-Indigenous CHWs working within Indigenous communities.	ICHWs (Indigenous child health workers) require support in relation to the cultural safety of the workplace, educational opportunities, collaboration with colleagues and peers, and professional mentorship, improvement of which can increase job satisfaction.	50%

^1^ Additional information about methodology sourced from Robinson (2007) [43]; ^2^ Additional information about methodology sourced from Duraisingam (2010) [44]; ^3^ Additional information about methodology sourced from Roche (2010) [21]. MMAT: Mixed Methods Appraisal Tool.

**Table 2:** Grey literature.

Author (Year)	Methods	Study Population and Response Rate	Focus	Relevant Findings	MMAT Score
Aboriginal and Torres Strait Islander Health Workforce Working Group (2017) [45] Australia	Qualitative Consensus	None. Working party consensus with input from Indigenous health stakeholders.	Framework to guide IHW workforce policy and planning.	Six strategies (with suggested actions) for a stronger workforce: improve recruitment and retention, improve skills and capacity, provide culturally-safe workplaces, increase number of health students, improve completion rates for health students, improve health workforce planning and policy.	N/A
Congress of Aboriginal and Torres Strait Islander Nurses and Midwives (2014) [46] Australia	Mixed methods Survey, yarning circles and forums	67 participants (57 CATSINaM Members (nurses or student nurses, approx. 28% of total membership) and 11 non-Members). Yarning Circles: unspecified. Forums: unspecified.	Proposed solutions for a revised mentoring program for Indigenous nurses.	Mentoring was identified as a priority strategy to improve retention among Indigenous nurses through the provision of cultural support and preceptoring relationships.	25%
Health Workforce Australia (2014) [47] Australia	Qualitative Interviews	13 Indigenous health leaders (3 CEOs, 3 middle management, 2 clinical management, 5 academics; 9 participants also current or former clinicians). Response rate: 65% of identified key informants.	Challenges faced by Indigenous health leaders and recommendations to support and develop current and future leaders.	Shortage of Indigenous health leaders causes high workloads and stress. Covert and systemic racism in the health system contributes to stress. Need for cultural competency for all health leaders. Need to improve overall legitimacy, credibility and support for Indigenous health workforce. Need for succession planning and mentoring to develop future Indigenous health leaders.	75%
Health Workforce Australia (2011) [48] Australia	Mixed methods Interviews, survey and focus groups	923 health professionals. Survey: 351 IHWs (response rate of 22–35%), 100 managers. Focus Groups: 264 IHWs, 100 managers, 25 health professionals. Interviews: 138 key informants.	How the IHW workforce can be strengthened.	Barriers that affect retention of IHWs include: low salary and salary inequities, lack of job security, burn-out, lack of respect and support, and limited career progression opportunities. Other challenges for IHWs include limited professional development opportunities, racism and lack of cultural security in the workplace and inadequate supervision. Enablers to retention: high job satisfaction, strong ties to the community and wanting to make a difference for the community. Effective retention strategies included: supportive management structure, respect from colleagues, Indigenous leadership in the health workforce, culturally safe workplace, flexible working conditions, and access to professional development.	75%
Health Workforce Australia (2011) [19] Australia	Mixed methods Interviews, survey and focus groups	1052 health professionals. Survey: 351 IHWs (response rate of 22–35%), 100 managers. Focus Groups: 264 IHWs, 100 managers, 25 health professionals. Interviews: 212 key informants (138 individuals in Phase 1 and 74 individuals in Phase 2).	Policies and strategies that aim to strengthen and sustain the IHW workforce.	Makes 27 recommendations to support and strengthen the IHW workforce including recommendations to improve retention (such as addressing salary inequities). Includes the HWA Health Service Toolkit (Appendix F) which provides actions that health services can undertake to support IHWs and address retention challenges.	75%

CATSINaM: Congress of Aboriginal and Torres Strait Islander Nurses and Midwives. HWA: Health Workforce Australia. N/A: Not applicable. This publication was not suitable for quality appraisal with the MMAT scoring system.

**Table 3:** Factors relating to retention of Indigenous Australians in the health workforce *.

Study Criterion	Structural	System	Organisational	Individual
Enablers	None	None	Co-worker support and peer mentorship (8) [19,34,38,39,40,42,46,48]. Culturally safe workplace (4) [41,42,47,48]. Access to clinical and cultural supervision (4) [37,40,42,48]. Professional development opportunities (4) [39,40,42,48]. Job security and adequate remuneration (2) [37,40].	Making a difference for Indigenous health (5) [39,41,42,47,48].
Barriers	Racism (5) [35,40,41,47,48]	Limited organisational funding and inadequate remuneration (8) [9,36,37,40,41,42,47,48]. Limited career pathways (2) [36,48].	Heavy workloads and demands (10) [9,35,36,37,38,40,41,42,47,48]. Lack of support from management and lack of mentoring (8) [34,35,37,38,41,46,47,48]. Lack of professional development opportunities (5) [9,36,37,38,41].	Proximity to community (8) [35,36,37,40,41,42,47,48].
Recommendations	None	Recognition of the Indigenous health professional role (8) [9,19,37,38,40,41,45,47]. Increased remuneration and salary parity (7) [9,19,37,39,40,41,48]. Work with educational systems to improve curriculum structure and facilitate career progression (4) [19,36,39,45].	Implement mentoring, clinical supervision and support systems (10) [9,19,34,35,37,39,41,42,45,46]. Embed cultural respect in the workplace (7) [19,34,36,37,41,45,47]. Professional development opportunities (7) [19,39,40,41,42,45,47]. Flexible working arrangements (3) [19,40,41].	None.

* Numbers in round brackets refer to number of relevant articles identifying this factor.

## Supplementary Materials

The following are available online at http://www.mdpi.com/1660-4601/15/5/914/s1, Search Strategies.
