# Supplementary material for: Factors Affecting the Retention of Indigenous Australians in the Health Workforce: A Systematic Review
Source: Int J Environ Res Public Health. 2018 May 4;15(5):914. doi: 10.3390/ijerph15050914 (PMC5981953; doi:10.3390/ijerph15050914)
Supplement: Supplementary file 1 [file ijerph-15-00914-s001.pdf]

Factors Affecting the Retention of Indigenous Australians in the Health Workforce: A Systematic Review

Search Strategies

Search Strategies for Peer-reviewed Studies

Database: PubMed

Search Date: 10 August 2017

1	oceanic ancestry group[mh] OR american native continental ancestry group[mh]
2	indigenous OR indigene* OR aborigin* OR “torres strait islander” OR “torres strait islanders” OR maori* OR polynesian* OR “pacific peoples” OR “native american” OR “native americans” OR “american indian” OR “american indians” OR amerind* OR alaskan* OR eskimo* OR “native hawaiian” OR “native hawaiians” OR “first nation” OR “first nations” OR inuit* OR metis OR “native canadian” OR “native canadians” OR “canadian indian” OR “canadian Indians”
3	1 or 2
4	Employment[mh] OR Health Personnel[mh] OR workplace[mh]
5	Worker* OR workforce OR staff OR personnel OR “healthcare providers” OR “Health Care Providers” OR “healthcare workers” OR “health care workers” OR “health workers”
6	4 or 5
7	Personnel turnover[mh]
8	Remain OR retain OR retention OR turnover OR barrier* OR enabler* OR facilitator*
9	7 or 8
10	3 and 6 and 9

Database: CINAHL Plus

Interface: EBSCOhost

Search Date: 10 August 2017

1	MH "Indigenous Peoples+"
2	indigenous OR indigene* OR aborigin* OR “torres strait islander” OR “torres strait islanders” OR maori* OR polynesian* OR “pacific peoples” OR “native american” OR “native americans” OR “american indian” OR “american indians” OR amerind* OR alaskan* OR eskimo* OR “native hawaiian” OR “native hawaiians” OR “first nation” OR “first nations” OR inuit* OR metis OR “native canadian” OR “native canadians” OR “canadian indian” OR “canadian Indians”
3	1 or 2
4	“Workforce” OR MH “Health Personnel+”

5	Worker* OR workforce OR staff OR personnel OR “healthcare providers” OR “Health Care Providers” OR “healthcare workers” OR “health care workers” OR “health workers”
6	4 or 5
7	MH “Personnel Turnover” OR MH “Personnel Retention”
8	Remain OR retain OR retention OR turnover OR barrier* OR enabler* OR facilitator*
9	7 or 8
10	3 and 6 and 9

Database: Informit Indigenous Collection

Search Date: 16 August 2017

1	Worker* OR workforce OR staff OR personnel OR “healthcare providers” OR “Health Care Providers” OR “healthcare workers” OR “health care workers” OR “health workers”
2	Remain OR retain OR retention OR turnover OR barrier* OR enabler* OR facilitator*
3	1 and 2

Database: Informit Health Collection

Search Date: 16 August 2017

1	indigenous OR indigene* OR aborigin* OR “torres strait islander” OR “torres strait islanders”
2	Worker* OR workforce OR staff OR personnel OR “healthcare providers” OR “Health Care Providers” OR “healthcare workers” OR “health care workers” OR “health workers”
3	Remain OR retain OR retention OR turnover OR barrier* OR enabler* OR facilitator*
4	1 and 2 and 3

Search Strategies for Grey Literature

Source: Health Organisation Websites

Where possible, websites were searched using relevant search terms such as 'retention', 'retain' and 'health workforce'.

Where a search interface did not exist, relevant publications where identified by scanning the list of publications and reports for each organisation and reviewing titles and summaries.

Health Organisation Website	Dates Searched	Publications Identified
Australian Indigenous Doctors Association (AIDA)	1 September 2017	0
Australian Institute of Health and Welfare (AIHW)	10 August 2017	1
Congress of Aboriginal and Torres Strait Islander Nurses and Midwives (CATSINaM)	1 September 2017	1
Health*InfoNet*	10 August 2017	1
Health Workforce Australia (HWA)	10 August 2017	5
Indigenous Allied Health Australia (IAHA)	1 September 2017	0
LIME (Leaders in Indigenous Medical Education) Resource Hub	31 August 2017, 1 September 2017	15
National Aboriginal and Torres Strait Islander Health Worker Association (NATSIWA)	1 September 2017	0

Source: Health*InfoNet* Endnote database

Searched using combinations of the following keywords – "Aboriginal" or "Indigenous" and "worker" or "workforce" on titles. This identified 23 publications.
